# Endoscopic management of a giant symptomatic duodenal lipoma causing partial obstruction

**DOI:** 10.1055/a-2875-0038

**Published:** 2026-06-01

**Authors:** Ankoor H. Patel, Petros C. Benias, Michael Ma, Srinivas S. Vasireddi, Arvind J. Trindade

**Affiliations:** 1 Division of GastroenterologyRutgers University School of MedicineNew BrunswickNew JerseyUnited States

Gastrointestinal lipomas are benign mesenchymal tumors arising from submucosal adipose tissue. Larger lesions, typically those exceeding 2 cm, are rare and carry a higher risk of complications, including bleeding, intussusception, and luminal obstruction 1 2 3 4 . Traditionally, surgery is the mainstay for large and symptomatic lesions.

**Fig. 1:**
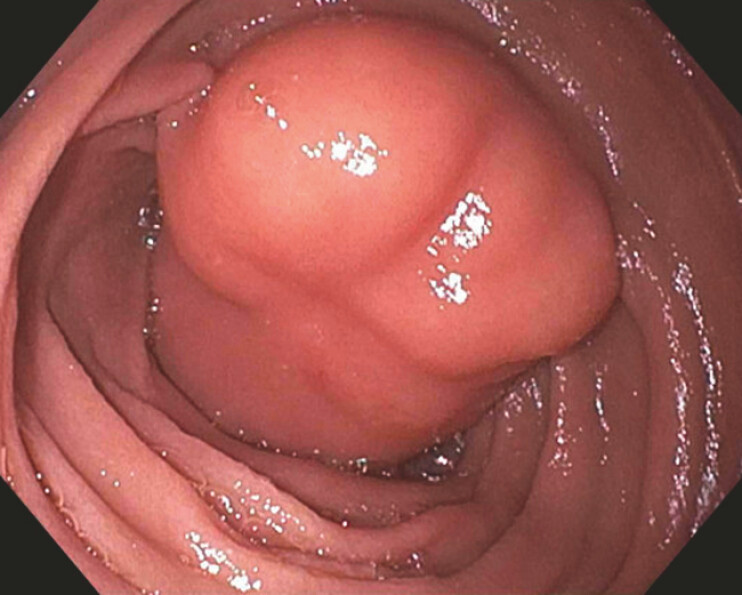
A duodenal subepithelial lesion occupying most of the lumen.

**Fig. 2:**
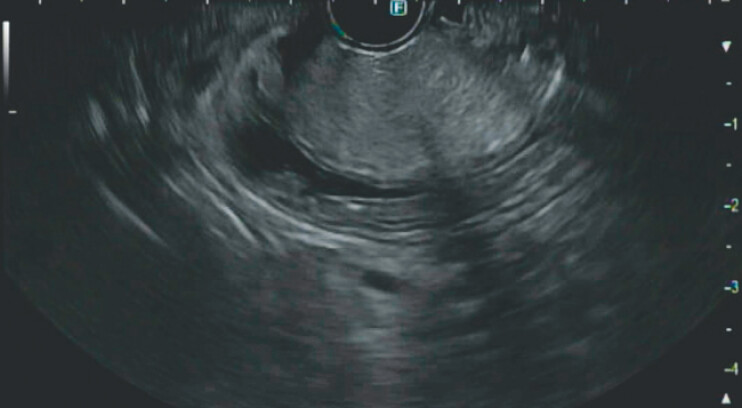
Endoscopic ultrasound showing a duodenal lipoma.

We present a 45-year-old woman with post-prandial abdominal pain and vomiting, early satiety, and a 2-month history of 15-pound weight loss. Computed tomography revealed a 6 cm duodenal lipoma. Esophagogastroduodenoscopy revealed a duodenal subepithelial lesion occupying most of the lumen ( Fig. 1 ). Endoscopic ultrasound demonstrated a submucosal lesion exceeding 6 cm in size arising from the third mucosal layer, with well-defined borders and a homogeneous, hyperechoic echotexture, confirming the diagnosis of lipoma ( Fig. 2 ). Endoscopic resection was initially considered. However, given the large size, this would require significant electrocautery current. Given the adipose content of lipomas, they are poor conductors of electrocautery. These technical issues, combined with the thin wall of the duodenum, made this technique extremely high-risk for perforation.

Before subjecting the patient to a high-risk endoscopic resection procedure or surgery, we decided to employ the endoscopic strangulation technique, which has been described for other subepithelial lesions (carcinoids and GIST) in the gastrointestinal tract 5 , but not for giant lipomas. Given its bilobar morphology, two endoloops were successfully placed around each lobe ( Video 1 ). After placement, mucosal ischemic changes were seen consistent with successful ligation. The patient reported melena, which resolved within 24 hours on day 10 post-procedure. She reported resolution of symptoms at day 12. Repeat endoscopy at 5 weeks confirmed complete resolution with a healthy, intact resection site and no residual lesion.

Video showing the endoscopic management of the duodenal lipoma.Video 1

In summary, we report a case of a symptomatic giant duodenal lipoma. To our knowledge, this is the first case of this rare entity, managed endoscopically.

Endoscopy_UCTN_Code_TTT_1AO_2AN
